# Analysis of uterine evacuation methods in postabortion care after implementation of a surveillance network (CLAP MUSA-Network) at a university hospital

**DOI:** 10.1371/journal.pone.0296009

**Published:** 2023-12-15

**Authors:** Nelio N. Veiga-Junior, Caroline Eugeni, Beatriz D. Kajiura, Priscilla B. F. Dantas, Caroline B. Trabach, Aline A. Junqueira, Carina C. Nunes, Luiz F. Baccaro

**Affiliations:** Department of Obstetrics and Gynecology, University of Campinas (UNICAMP) School of Medicine, Campinas, SP, Brazil; National Academy of Medical Sciences, NEPAL

## Abstract

**Background:**

Management of uterine evacuation is essential for increasing safe abortion care. Monitoring through surveillance systems tracks changes in clinical practice and provides information to improve equity in abortion care quality.

**Objective:**

This study aimed to evaluate the frequency of manual vacuum aspiration (MVA) and medical abortion (MA), and identify the factors associated with each uterine evacuation method after surveillance network installation at a Brazilian hospital.

**Methods:**

This cross-sectional study included women admitted for abortion or miscarriage to the University of Campinas Women’s Hospital, Brazil, between July 2017 and November 2020. The dependent variables were the use of MVA and MA with misoprostol. The independent variables were the patients’ clinical and sociodemographic data. The Cochran–Armitage, chi-square, and Mann–Whitney U tests, as well as multiple logistic regression analysis, were used to compare uterine evacuation methods.

**Results:**

We enrolled 474 women in the study, 91.35% of whom underwent uterine evacuation via uterine curettage (78.75%), MVA (9.46%), or MA (11.54%). MVA use increased during the study period (Z = 9.85, p < 0.001). Admission in 2020 (odds ratio [OR] 64.22; 95% confidence interval [CI] 3.79–1086.69) and lower gestational age (OR 0.837; 95% CI 0.724–0.967) were independently associated with MVA, whereas the only factor independently associated with MA was a higher education level (OR 2.66; 95% CI 1.30–5.46).

**Conclusion:**

MVA use increased following the installation of a surveillance network for good clinical practice. Being part of a network that encourages the use of evidence-based methods provides an opportunity for healthcare facilities to increase access to safe abortions.

## Introduction

Estimates suggest that 208 million women worldwide become pregnant every year and approximately 25% of all pregnancies end in abortion [1]. One in two abortions, mostly in developing countries, is unsafe, leading to approximately 47000 yearly maternal deaths due to complications [1–3]. In Brazil, despite the National Abortion Survey showing a drop in abortion hospitalization rates [4], 200 women die annually from unsafe abortions. Furthermore, 20% of women up to the age of 40 have had an illegal abortion, half of whom were subsequently hospitalized for abortion-related complications [5–7]. Restrictive laws only permitting pregnancy termination in cases of sexual violence, risk of maternal death, or fetal anencephaly limit the provision of safe abortion, allowing avoidable morbidity and mortality to persist [7,8].

Improving women’s access to postabortion care methods is recommended by the World Health Organization (WHO) guidelines for reducing unsafe abortion [1,9,10]. The management of uterine evacuation is essential in comprehensive abortion care and reduces abortion-related complications. Further, it minimizes the costs associated with treating unsafe abortion complications [1,9,11,12]. Therefore, efforts to replace uterine dilatation and curettage (D&C), which is associated with a higher risk of uterine perforation and intrauterine synechiae, with alternative methods are recommended [10,13]. Medical abortion (MA) using pharmacological drugs such as mifepristone or misoprostol is safe and effective, is associated with fewer complications, avoids surgery-related risks, and is feasible in low-resource settings [9,14]. Additionally, the recommended surgical uterine evacuation technique is manual vacuum aspiration (MVA), which is safer, faster, less painful, and associated with fewer complications than D&C [15].

The method of uterine evacuation highlights the quality of a healthcare facility’s abortion care [16–18]. Respectful services and women-centered counseling provide positive experiences and reduce adverse abortion-related outcomes. In the decision-making process, women choose MA four times more frequently than other methods. For those who prefer surgical methods, MVA leads to fewer side effects and greater satisfaction than sharp curettage [15]. Monitoring these indicators for safe abortions enables assessment of disparities in the quality of care provided to these women.

Ensuring a standard in abortion healthcare services may identify which evidence-based practices should be prioritized for data collection [10]. Surveillance systems can improve the quality of care and help countries monitor and evaluate programs aimed at preventing unplanned pregnancies as well as maternal morbidity and mortality. Moreover, abortion surveillance programs can track changes in clinical practice patterns and provide the data necessary to inform health policies [10,16,18]. In a previous study, we demonstrated how the use of a surveillance system increased contraceptive use before discharge in women hospitalized for abortion complications [19].

In Brazil, even with a decrease in abortions, almost 70% of all pregnancies are unplanned [4,20]. Restrictive laws increase the burden of unsafe abortions, which remain an important health issue [21]. The method of uterine evacuation impacts abortion safety provision, and monitoring clinical practices over time is necessary to improve postabortion care and foster improved public policies [1,10,19]. Therefore, this study aimed to evaluate the frequency of MVA and MA use in a hospital in Brazil and identify factors associated with the method of uterine evacuation after the implementation of a surveillance network.

## Methods

### MUSA network

The multicentric MUSA (mujeres en situación de aborto) Network was created by the Latin American Center for Perinatology (CLAP) to improve care for women undergoing abortions in Latin America and the Caribbean [22]. The network includes several hospitals, classified as sentinel centers, that periodically send pregnancy data for registration in the Perinatal Computerized System (SIP), software developed by CLAP that aids in recording data related to pregnancy and epidemiological monitoring. Our institution, the University of Campinas Women’s Hospital (UNICAMP), is a tertiary referral hospital located in an urban setting in southeast Brazil. The hospital handles cases of pregnancy-related complications in municipalities within the region and sees an average of 250 births and 20 cases of first-trimester pregnancy loss per month. Our institution has been a sentinel center of the CLAP MUSA-Network since July 2017. The National Research Ethics Committee approved data collection at the hospital prior to the commencement of the study (approval number: 62778316.6.1001.5404).

The sentinel centers of the CLAP MUSA-Network regularly provide data on maternal morbidity in early pregnancy loss, uterine evacuation methods for termination, incidence of termination-related complications, incidence of preoperative antibiotic use, and contraceptive prescription before hospital discharge. SIP enables epidemiological monitoring and comparisons between centers over time. Additionally, representatives from each center hold regular online meetings to discuss the collected data, conduct scientific talks on the health of women undergoing abortions, and encourage good clinical practice for safe abortions. The CLAP MUSA-Network protocol and specific methodological details have been published elsewhere [23].

### Study design and data collection

This cross-sectional prospective study was conducted between July 2017 and November 2020. We included all women admitted to our institution for an abortion for any legal reason and those considered to be in postabortion circumstances (including miscarriage and early pregnancy loss). The exclusion criteria were bleeding during pregnancy with no confirmed abortion and ectopic or molar pregnancies. Data recorded in SIP are based on information collected through face-to-face interviews with women during hospitalization and a review of the data in their medical records. All participants provided written informed consent. Minors and their legal guardians provided informed assent and consent, respectively. The Research Ethics Committee of our institution (CEP UNICAMP) approved the study protocol (approval number: 93060618.9.1001.5404).

In all abortion cases, MA was performed using vaginal administration of misoprostol, since mifepristone use has not yet been approved in Brazil. Further, surgical approaches comprised MVA and D&C. All medical staff members were trained to perform each uterine evacuation method. At the time of hospitalization, the women received information about all the methods, although the approach used was determined by a healthcare provider.

The dependent variables for analysis were each uterine evacuation method, namely MVA and MA with misoprostol only (when there was no need for further uterine evacuation). The independent variables were age, education, marital status, medical history, number of pregnancies, number of births, number of abortions, body mass index (BMI), active smoking, illegal drug use, alcohol use, planned pregnancy, pregnancy resulting from contraceptive failure, date of admission for abortion, legal abortion, gestational age, presence of complications, and other admission data.

### Power calculation

This study used a convenience sample of all women who fulfilled the selection criteria and provided informed consent between 07/01/2017 and 11/16/2020. We calculated the statistical power of the study using the estimate in a descriptive study with a categorical variable, setting the alpha level of significance or type I error at 5% and the sample error at 5%. The power of the sample to estimate the prevalence of MVA and MA were 99.4% and 97.3%, respectively.

### Statistical analyses

First, a descriptive analysis of the data was performed. The continuous variables are presented as the mean and standard deviation or the median and interquartile range. Relative frequencies were calculated for the categorical variables. The Cochran–Armitage trend test was performed with quarterly analyses to assess whether the rates of MVA and MA use changed over time. Thereafter, chi-square (categorical variables) and Mann–Whitney U tests (continuous variables) were conducted to compare the clinical and sociodemographic factors associated with performing MVA and MA. Finally, to evaluate the factors independently associated with MVA and MA, two multiple logistic regression models were constructed using stepwise criteria for variable selection. The significance level was set at 5% for all tests, and all analyses were performed using the SAS System for Windows version 9.2 (SAS Institute Inc., Cary, NC, USA).

## Results

During the study period, 474 women with a mean age of 30.01 (± 7.48) years and median age of 30 (12–48) years were enrolled. The mean gestational age was 11.03 (± 3.56) weeks, and 30.38% of the women had not previously been pregnant. Most women did not use contraception (75.5%), and 69.85% of the pregnancies were unplanned. Of the 474 cases, 67 (14.1%) were legal abortions, and 66 of the 67 (98.5%) pregnancies resulted from sexual violence. Details of the patients’ clinical and sociodemographic characteristics are presented in **Table 1**.

**Table 1:** Clinical and sociodemographic characteristics (n = 474).

	n	%
**Age** ^**a**^		
< 20 years	31	6.55
20–29 years	204	43.13
30–39 years	182	38.48
40–49 years	56	11.84
**Education** ^**b**^		
None	3	0.64
Primary	84	17.83
Secondary	288	61.15
Tertiary	96	20.38
**Marital status** ^**c**^		
Married	183	39.61
Cohabiting	87	18.83
Single	146	31.60
Other	46	9.96
**Previous abortion** ^**d**^		
0	297	68.28
1	98	22.53
2	26	5.98
3	10	2.30
4	1	0.23
**Planned pregnancy** ^**e**^		
Yes	329	69.85
No	142	30.15
**Contraceptive use** ^**f**^		
None	352	75.54
Hormonal	31	6.65
Barrier	8	1.72
IUD	69	14.81
Emergency	4	0.86
Natural	2	0.43
**Legal abortion**		
Yes	67	14.14
No	407	85.86
**Sexual violence** ^**g**^		
Yes	66	13.95
No	407	86.05

Abbreviations: IUD, intrauterine device.

^a–g^Missing data (a = 1; b = 1; c = 12; d = 39; e = 3; f = 8; g = 1).

**Fig 1:**
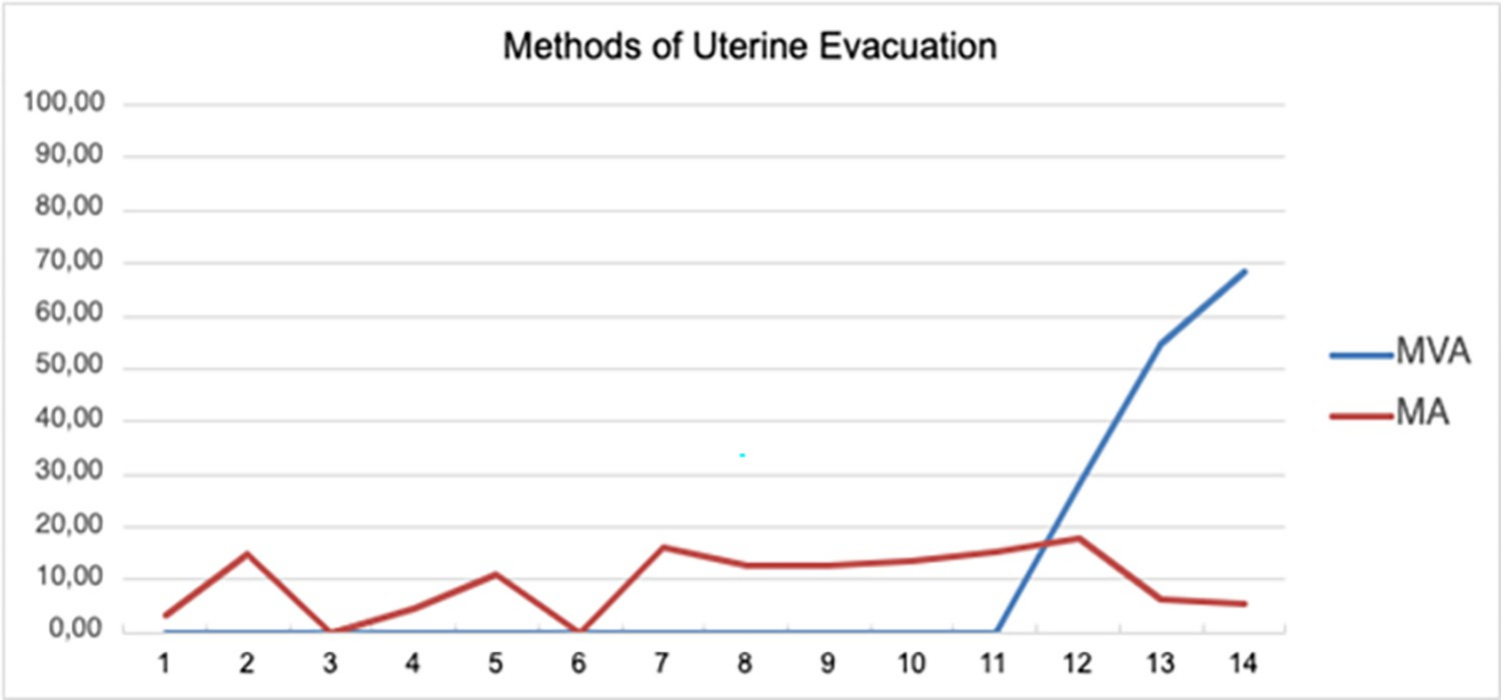
Methods of uterine evacuation between July 2017 and November 2020 by trimestral period. **Captions:** MVA- manual vacuum aspiration; MA- medical abortion Cochran-Armitage test: MVA: Z = 9.85, P <0.001; MA: Z = 1.35; P = 0.178.

During the evaluation period, 433 (91.35%) women underwent uterine evacuation. The most common method was D&C, which was performed in 341 patients (78.75%). MVA was performed in 41 (9.46%) women, and MA using misoprostol was administered to 50 women (11.54%). At least one complication was observed in 24 of the 474 (5.06%) women, all of whom underwent D&C. Over the course of the surveillance period, we observed a significant increase in the use of MVA (Cochran–Armitage test: Z = 9.85; p < 0.001); however, no difference in the use of MA was observed (Z = 1.35; p = 0.178) (**Fig 1**).

Considering the factors associated with MVA, the procedure was performed more frequently in women with a lower education level (p = 0.04), those admitted in 2020 (p < 0.01), and those with no previous pregnancies (p < 0.01), lower BMI (p < 0.01), lower gestational age (p = 0.03), shorter symptom duration (p = 0.02), and higher hemoglobin levels (p = 0.04). In the multiple logistic regression analysis, admission in 2020 (odds ratio [OR] 64.22; 95% confidence interval [CI] 3.79–1086.69) and lower gestational age (OR 0.837; 95% CI 0.724–0.967) were independently associated with MVA. Regarding MA, women with a higher education level underwent this procedure more frequently (p = 0.03). Furthermore, the regression analysis revealed that a higher education level was the only factor independently associated with MA (OR 2.66; 95% CI 1.30–5.46) (**Table 2**).

**Table 2:** Factors associated with uterine evacuation method (n = 381)^a^.

Variable	OR (95% CI)	p-value
**Manual vacuum aspiration**		
**Year of admission**		
2017	Reference	
2018	1.00 (0.99–1.01)	1.000
2019	1.00 (0.99–1.01)	1.000
2020	64.22 (3.79–1086.69)	< 0.001
**Gestational age**	0.837 (0.724–0.967)	0.016
**Medical abortion**		
**Education**		
None	2.21 (0.10–47.69)	0.675
Primary	1.01 (0.36–2.83)	0.984
Secondary	Reference	
Tertiary	2.66 (1.30–5.46)	0.008

Abbreviations: CI, confidence interval; OR, odds ratio.

^a^Multiple logistic regression analysis by stepwise variable selection to determine factors associated with the uterine evacuation method. Cases with missing variables were not included in the multiple regression analysis.

## Discussion

The present study describes the uterine evacuation methods performed at a university hospital with an established surveillance network that encourages safe abortion practices (the CLAP MUSA-Network). During the observation period, MVA use increased, while the rate of MA remained unchanged. Women admitted in 2020 and those with a lower gestational age had an increased likelihood of undergoing MVA. In contrast, a higher level of education was associated with MA use.

In surgical abortion, replacing D&C with MVA is the key to preventing unsafe abortions [1,16]. Nevertheless, D&C is the second most frequently performed obstetric procedure in Brazilian public health facilities after vaginal delivery [20]. According to a national mixed methods study, only 45% of women who used legal abortion services underwent MVA [24]. Furthermore, several initiatives to replace D&C have not achieved positive results, differing from our findings. No overall improvement in MVA frequency was observed in Honduras and Malawi’s postabortion care, mostly because of the reluctance of healthcare providers to abandon D&C [25,26]. The present analysis shows a significant trend toward increased MVA use following implementation of the MUSA Network. Although the rate of MVA use was relatively low (9.46%) for most of the evaluation period, we observed a considerable increase in the final 6 months of the study, with the rate exceeding 60% in the last 3 months of 2020. The CLAP MUSA-Network promoted regular scientific meetings with other sentinel centers, reinforcing the importance of safe abortions and improving medical staff training. Nevertheless, we observed a relatively long period before the routine use of MVA was established in the hospital. We believe that this is because the hospital administrative team should also have been made aware of the use of safer uterine evacuation techniques. The meetings promoted by the CLAP MUSA Network were essential for raising awareness among the health unit’s management team to ensure the acquisition of hospital supplies necessary to routinely perform MVA on a permanent basis. This finding supports the idea that monitoring clinical care promotes and improves comprehensive abortion care [23,27]. Similar to our study, institutions in Bangladesh have also used monitoring to evaluate the progress achieved in replacing D&C with MVA and MA [28]. Additionally, the routine Abortion Surveillance program in the United States reports on the use of different methods to analyze the safety of abortion practices and has fostered a reduction in unplanned pregnancies [27].

MA is considered an effective alternative to surgical procedures, with a success rate of 75–90% [28,29]. In the present study, MA was performed in 11.54% of the patients, and no increase in its use was observed during the study period. In the United States, approximately 40% of all abortions in 2018 were performed exclusively using medication [27]. In Brazil, mifepristone is not available, and misoprostol is only used for uterine emptying in hospitalized patients [8]. Compared to surgical procedures, MA takes longer to complete and requires a longer hospital stay; thus, it may not be the first choice for women or medical professionals [1,12].

Follow-up after MA is based on the self-recognition of signs and symptoms [6]. Healthcare professionals might believe it is safer to recommend MA to women who may more readily recognize warning signs, preemptively seek medical services, and have a better understanding of the choices available [30]. In our study, approximately 20% of the women had a higher education level and a 2.3-times greater chance of receiving MA. Although we did not evaluate the decision-making process regarding the abortion method, other studies have reported similar results. Even when the procedures were performed by the same organization and professional, women who chose MA had a higher level of education [31,32].

This study has some limitations. First, the cross-sectional nature of the study means that a cause-and-effect relationship could not be established. Second, differentiating between provoked and spontaneous abortions was not possible, except in cases of legal induction. Regarding MA, the use of misoprostol in Brazil is restricted to the hospital setting, which prevents dispensation for home use. It was not possible to consider the influence of the misoprostol dosage used during the study period. Finally, the patients were only analyzed during hospitalization and were not followed up after discharge. Although the uterine evacuation methods are discussed with patients, the final decision lies with the responsible doctor. Nevertheless, we believe that our results are valid. The implementation of the CLAP MUSA-Network for the surveillance and implementation of good clinical practices has ensured the monitoring of clinical decisions inside institutions and promoted healthcare providers’ ongoing education, leading to a reduction in the use of D&C. The provision of safe abortion methods is central to the WHO and FIGO goal for equitable access to abortion care and improved women’s reproductive health [10,16].

Being part of a network that encourages the use of evidence-based clinical practices provides an opportunity for healthcare facilities to set priorities, evaluate outcomes, and implement policy changes to improve safe abortion access. Continuous monitoring of therapeutic indicators and medical education can help reduce abortion-related morbidities and mortality, and address the healthcare needs and rights of women.
